# Supplementary material for: Global Downregulation of Penicillin Resistance and Biofilm Formation by MRSA Is Associated with the Interaction between Kaempferol Rhamnosides and Quercetin
Source: Microbiol Spectr. 2022 Nov 10;10(6):e02782-22. doi: 10.1128/spectrum.02782-22 (PMC9769653; doi:10.1128/spectrum.02782-22)
Supplement: Supplemental file 1 — Supplemental material. Download spectrum.02782-22-s0001.pdf, PDF file, 1.0 MB [file spectrum.02782-22-s0001.pdf]

Table S1 The primers used for (RT)-PCR amplification

Genes	Forward sequence (5'-3')	Reverse sequence (5'-3')	Expected product fragment size (bp)	Reference
16S rRNA	CGGTCCAGACTCCTACGGGAGGCAGCA	GCGTGGACTACCAGGGTATCTAATCC	481	(1)
agrA	TCTCACAGACTCATTGCCCATT	GGCGATTGACGACAAAGCT	121	In this study
atlA	AACAGCACCAACGGATTAC	CATAGTCAGCATAGTTATTCATTG	131	In this study
blaZ	GATAAGAGATTTGCCTATGC	GCATATGTTATTGCTTGACC	533	In this study
cidA	TGTACCGCTAACTTGGGTAGAAGAC	CGGAAGCAACATCCATAATACCTAC	101	In this study
clfA	ATTGGCGTGGCTTCAGTGCT	CGTTTCTTCCGTAGTTGCATTTG	288	(1)
clfB	ACATCAGTAATAGTAGGGGCAAC	TTCGCACTGTTTGTGTTTGCAC	203	(1)
coa	GTAGATTGGGCAATTACATTTTGGAGG	CGCATCTGCTTTGTTATCCCATGT	117	In this study
crtN	TGACGTCACTTGTGCAAGG	GAGCCATGCCATACATACCG	222	In this study
ebps	CATCCAGAACCAATCGAAGAC	AGTTACATCATCATGTTTATCTTTTG	180	(2)
fnbA	CATAAATTGGGAGCAGCATCA	ATCAGCAGCTGAATTCCCATT	128	(1)
hla	GGTTTAGCCTGGCCTTCAGC	ACCAGTAACATTACCGTTGAATCCA	150	In this study
hld	TAATTAAGGAAGGAGTGATTTCAATG	TTTTTAGTGAATTTGTTCACTGTGTC	100	In this study
icaA	ACACTTGCTGGCGCAGTCAA	TCTGGAACCAACATCCAACA	188	(1)
lrgA	CTGGTGCTGTTAAGTTAGGCG	GTGACATAGCCAGTACAAAT	190	(3)
luxS	CGGACTACATTCATTAGAACATT	TTACAAGCAGGCACTTCA	198	In this study
mecA	GCAATCGCTAAAGAACTAAG	GGGACCAACATAACCTAATA	220	(1)
psmα	TATCAAAAGCTTAATCGAACAATTC	CCCCTTCAAATAAGATGTTCATATC	176	(4)
rnaIII	TAAACATCCCAACTTGCCAGA	ATCCAAATACAATGCCCCAAT	205	In this study
sarA	GTTATCAATGGTCACTTATGC	CTTGTGGTTGTTTGTAGTTT	164	In this study
sigB	AGTGTTAGAAGCAATGGAAATG	CGATACGCTCACCTGTCTCT	251	In this study
sspA	CGATCGTCACCAAATCACAGA	TGCGTAGCATCTACGACGTGT	152	In this study
vwb	GGTACCAATGCCTACAGTTG	GTGATTGATGCGTTGTTGTG	116	In this study

Table S2 Functional roles of genes in biofilm formation and antibiotic resistance

Genes	Proteins	Functions involved in biofilm formation and *antibiotic resistance*	References
atlA	Autolysin A	Adhesion to hydrophilic and hydrophobic polystyrene surfaces; Mureiun hydrolysis, causing release of genomic DNA, contributes to initial attachment and biofilm accumulation; *Mureiun hydrolysis increases penicillin,vancomycin, and rifampin susceptibility*.	(5-9)
blaZ	Penicillinase	*Penicillin hydrolysis contributes to penicillin resistance.*	(10)
cidA	CidA protein	Positive regulating murein hydrolysis contributes to release of genomic DNA, involved in bacterial initial attachment and biofilm multiplication; *Increased murein hydrolysis reduces penicillin,vancomycin, and rifampin resistance.*	(7, 8)
clfA	Clumping factor A	Adhesion to fibrinogen contributes to initial adherence in biofilm formation	(11)
clfB	Clumping factor B	Adhesion to fibrinogen contributes to initial adherence in biofilm formation; Intercellular adhesion promotes accumulation of biofilm formation.	(11)
coa	Coagulase	Insoluble fibrin, produced as a result of coagulase activity, can function as a biofilm scaffold, allowing *S. aureus* to accumulate on human plasma-coated surfaces.	(12)
crtN	Dehydrosqualene desaturase	Staphyloxanthin protects bacterial cells from the damage of oxidative stress and host neutrophil-based killing, and thereby facilitates biofilm formation.	(13, 14)
ebpS	Elastin binding protein	Binding to elastin contributes to initial adherence in biofilm formation.	(15, 16)
fnbA	Fibronectin-binding protein A	Adhesion to fibronectin contributes to initial adherence in biofilm formation; intercellular adhesion promotes accumulation of biofilm formation.	(17, 18)

to be continued.

**Genes**	**Proteins**	**Functions involved in biofilm formation and antibiotic resistance**	**References**
hla	α-hemolysin	Alpha-toxin contributes to *S. aureus* mucosal biofilm formation while causing significant disruption to the mucosa; α-toxin plays a role in facilitating bacterial cell-to-cell interactions during biofilm formation on abiotic surfaces.	(19)
hld	δ-hemolysin	Surfactant activity contributes to biofilm dispersal and bacterial dissemination.	(20)
icaA	PIA/PNAG	Bacterial adhesion to biomaterial surfaces and intercellular adhesion contribute to initial adherence and accumulation of biofilm formation.	(21)
lrgA	LrgA protein	Negative regulating murein hydrolysis, reduced releasing of genomic DNA represses bacterial initial attachment and biofilm multiplication; *Reduced murein hydrolysis increases penicillin,vancomycin, and rifampin resistance.*	(7, 9)
mecA	Penicillin-binding protein 2a	*β-lactam resistance*; PIA-independent biofilm formation by *S. aureus*.	(18, 22)
psmα	Phenol-soluble modulin α	Surfactant activity contributes to biofilm dispersal and bacterial dissemination.	(11, 20)
sspA	Serine protease	Protein degradation contributes to biofilm dispersal and bacterial dissemination.	(20, 23)
vwb	von Willebrand factor-binding protein	Activation of prothrombin to form *S. aureus*-fibrin-platelet aggregates.	(24)

Table S3 Molecular docking affinity (kcal/mol) between compounds identified from *C. ambrosioides* L. and proteins of *S. aureus*

Compounds	Protein (PDB ID)		
	**RsbU (2J6Y)**	**SarA (2FNP)**	**AgrA (4G4K)**
Naringin dihydrochalcone	-9.0	-7.5	-9.6
Naringin	-8.9	-7.7	-8.6
Rutin	-8.3	-8.2	-9.3
Kaempferol-3,7-dirhamnoside	-8.1	-7.4	-10.1
Kaempferol-3-o-apigenin-7-o-rhamnoside	-8.7	-7.2	-9.6
Syringaresinol	-6.8	-6.7	-8.1
Kaempferol-3-o-acetylapigenin-7-o-rhamnoside	-8.1	-7.6	-9.6
Kaempferol-7-o-rhamnoside	-8.4	-7.5	-9.5
Quercetin	-7.7	-7.6	-8.1
Kaempferol-3-rutinoside	-8.9	-7.4	-9.5

PDB ID, Protein Data Bank identification.

Figure S1 Anti-biofilm activity of different extracts from *C. ambrosioides* L.

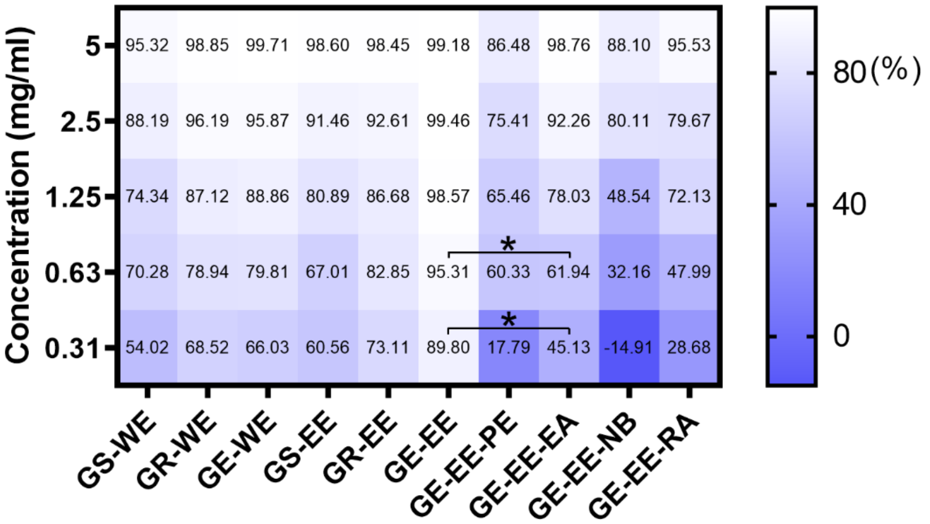

GE, the ear of *C. ambrosioides* L. originated from Guangxi, China; GR, the root of *C. ambrosioides* L.; GS, the stem of *C. ambrosioides* L.; EE, ethanol extracts; WE, water extracts; EA, fractional extraction with ethyl acetate; NB, fractional extraction with n-butanol; PE, fractional extraction with petroleum ether; RA, the residual aqueous layer after fractional extraction. *, significant at the level of 0.05 (2-tailed).

Figure S2 Photographs of antibacterial activity of different extracts from *C. ambrosioides* L.

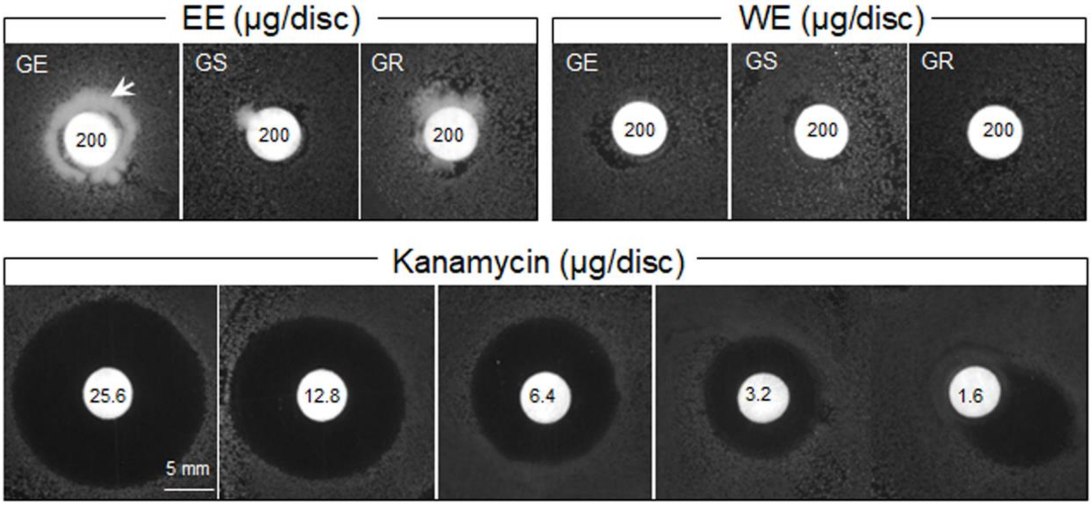

GE, the ear of *C. ambrosioides* L. originated from Guangxi, China; GR, the root of *C. ambrosioides* L.; GS, the stem of *C. ambrosioides* L.; EE, ethanol extracts; WE, water extracts; White arrow indicate markedly thickened bacterial lawn around disc.

Figure S3 Heat map of anti-biofilm effects of qurecetion in combination with penicillin G

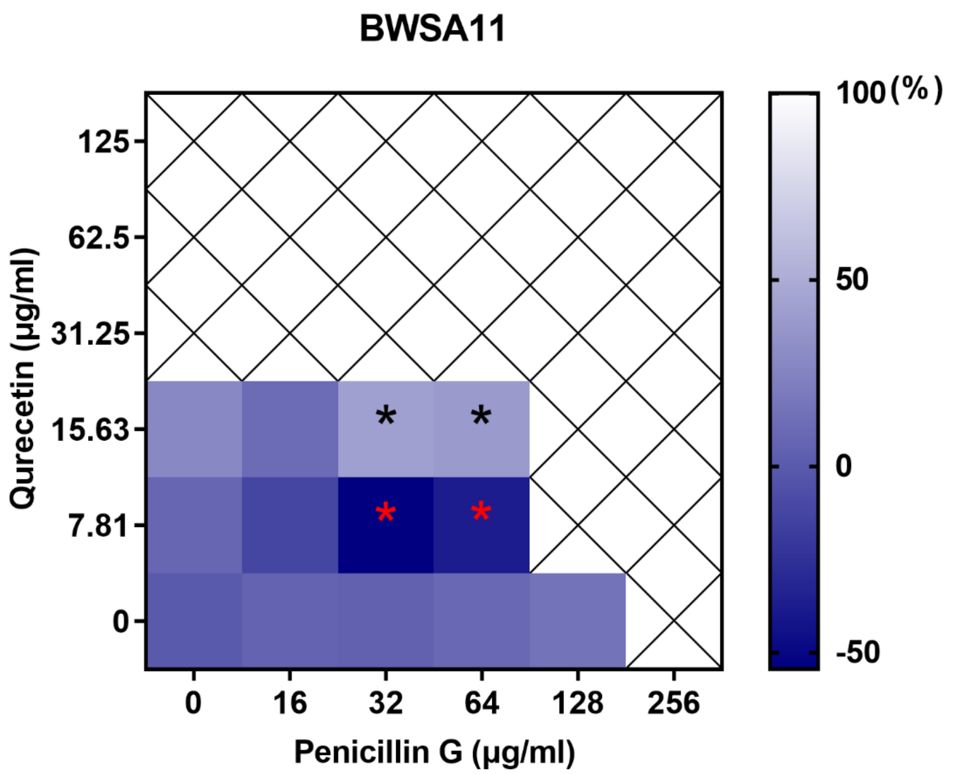

Not determined, the biofilm formation of the area that the bacterial inhibition rate is over 95% was not calculated. Black asterisks indicate significant decreases at a *P* value of 0.05 compared to control. Red asterisks indicate significant increases at a *P* value of 0.05 compared to control and either of the two parallel single-agent groups. All data are presented as means of three biological replicates.

Figure S4 Photographs of hemolytic activity of *S. aureus* strains in the presence of GE-EE

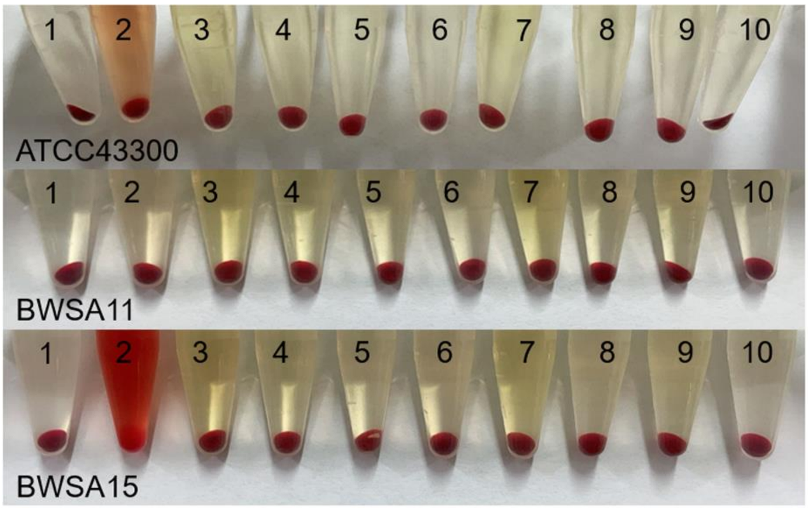

1, negative control without bacterial cells and in the absence of GE-EE; 2, positive control with bacterial cells in the absence of GE-EE; 3~6, groups without bacterial cells and in the presence of GE-EE at concentration of 2.5, 1.25, 0.625, and 0.3125 mg/ml, respectively; 7~10, groups with bacterial cells and in the presence of GE-EE at concentration of 2.5, 1.25, 0.625, and 0.3125 mg/ml, respectively.

Figure S5 Photographs of PIA production by *S. aureus* strains in the presence of GE-EE

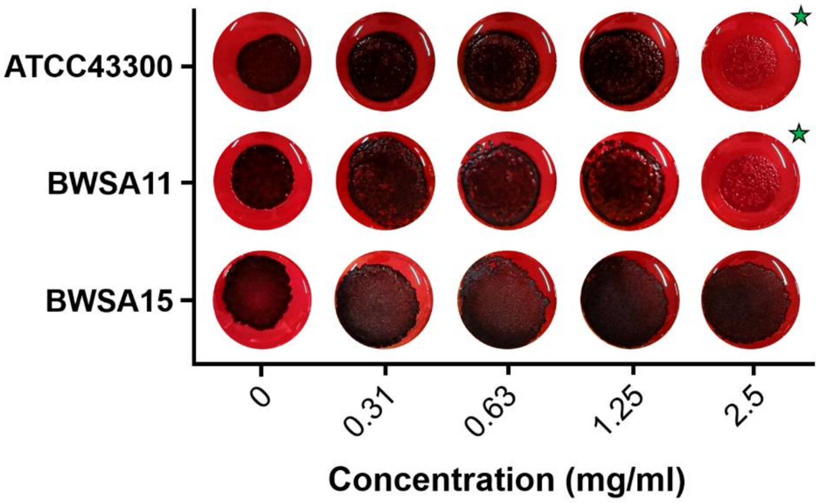

Black and brown lawns formed on Congo red agar indicate that bacteria produced PIA, and red lawns formed indicate bacteria did not produce PIA or less produce. Green star, significantly reduced production of PIA compared to control.

Reference

1. He X, Li S, Yin Y, Xu J, Gong W, Li G, Qian L, Yin Y, He X, Guo T, Huang Y, Lu F, Cao J. 2019. Membrane Vesicles Are the Dominant Structural Components of Ceftazidime-Induced Biofilm Formation in an Oxacillin-Sensitive MRSA. Frontiers in Microbiology 10.
2. Atshan SS, Nor Shamsudin M, Sekawi Z, Lung LTT, Hamat RA, Karunanidhi A, Mateg Ali A, Ghaznavi-Rad E, Ghasemzadeh-Moghaddam H, Chong Seng JS, Nathan JJ, Pei Pei C. 2012. Prevalence of Adhesion and Regulation of Biofilm-Related Genes in Different Clones of *Staphylococcus aureus*. Journal of Biomedicine and Biotechnology 2012:976972.
3. Liu G, Xiang H, Tang X, Zhang K, Wu X, Wang X, Guo N, Feng H, Wang G, Liu L, Shi Q, Shen F, Xing M, Yuan P, Liu M, Yu L. 2011. Transcriptional and functional analysis shows sodium houttuyfonate-mediated inhibition of autolysis in *Staphylococcus aureus*. Molecules 16:8848-65.
4. Li M, Cheung GY, Hu J, Wang D, Joo HS, Deleo FR, Otto M. 2010. Comparative analysis of virulence and toxin expression of global community-associated methicillin-resistant *Staphylococcus aureus* strains. J Infect Dis 202:1866-76.
5. Sugai M, Yamada S, Nakashima S, Komatsuzawa H, Matsumoto A, Oshida T, Suginaka H. 1997. Localized perforation of the cell wall by a major autolysin: *atl* gene products and the onset of penicillin-induced lysis of *Staphylococcus aureus*. J Bacteriol 179:2958-62.
6. Takahashi J, Komatsuzawa H, Yamada S, Nishida T, Labischinski H, Fujiwara T, Ohara M, Yamagishi J, Sugai M. 2002. Molecular characterization of an atl null mutant of *Staphylococcus aureus*. Microbiol Immunol 46:601-12.
7. Rice KC, Nelson JB, Patton TG, Yang SJ, Bayles KW. 2005. Acetic acid induces expression of the *Staphylococcus aureus cidABC* and *lrgAB* murein hydrolase regulator operons. J Bacteriol 187:813-21.
8. Rice KC, Firek BA, Nelson JB, Yang SJ, Patton TG, Bayles KW. 2003. The *Staphylococcus aureus cidAB* operon: evaluation of its role in regulation of murein hydrolase activity and penicillin tolerance. J Bacteriol 185:2635-43.
9. Groicher KH, Firek BA, Fujimoto DF, Bayles KW. 2000. The *Staphylococcus aureus lrgAB* operon modulates murein hydrolase activity and penicillin tolerance. J Bacteriol 182:1794-801.
10. Livermore DM. 2000. Antibiotic resistance in staphylococci. Int J Antimicrob Agents 16 Suppl 1:S3-10.
11. França A, Gaio V, Lopes N, Melo L. 2021. Virulence Factors in Coagulase-Negative Staphylococci. Pathogens 10:170.
12. Zapotoczna M, McCarthy H, Rudkin JK, O'Gara JP, O'Neill E. 2015. An Essential Role for Coagulase in *Staphylococcus aureus* Biofilm Development Reveals New Therapeutic Possibilities for Device-Related Infections. J Infect Dis 212:1883-93.
13. Liu CI, Liu GY, Song Y, Yin F, Hensler ME, Jeng WY, Nizet V, Wang AH, Oldfield E. 2008. A cholesterol biosynthesis inhibitor blocks *Staphylococcus aureus* virulence. Science 319:1391-4.
14. Clauditz A, Resch A, Wieland KP, Peschel A, Gotz F. 2006. Staphyloxanthin plays a role in the fitness of *Staphylococcus aureus* and its ability to cope with oxidative stress. Infect Immun

74:4950-3.

15. Foster TJ. 2019. The MSCRAMM Family of Cell-Wall-Anchored Surface Proteins of Gram-Positive Cocci. Trends Microbiol 27:927-941.

16. Downer R, Roche F, Park PW, Mecham RP, Foster TJ. 2002. The elastin-binding protein of *Staphylococcus aureus* (EbpS) is expressed at the cell surface as an integral membrane protein and not as a cell wall-associated protein. J Biol Chem 277:243-50.

17. O'Neill E, Pozzi C, Houston P, Humphreys H, Robinson DA, Loughman A, Foster TJ, O'Gara JP. 2008. A novel *Staphylococcus aureus* biofilm phenotype mediated by the fibronectin-binding proteins, FnBPA and FnBPB. J Bacteriol 190:3835-50.

18. Cortes MF, Beltrame CO, Ramundo MS, Ferreira FA, Figueiredo AM. 2015. The influence of different factors including *fnbA* and *mecA* expression on biofilm formed by MRSA clinical isolates with different genetic backgrounds. Int J Med Microbiol 305:140-7.

19. Anderson MJ, Lin YC, Gillman AN, Parks PJ, Schlievert PM, Peterson ML. 2012. Alpha-toxin promotes *Staphylococcus aureus* mucosal biofilm formation. Front Cell Infect Microbiol 2:64.

20. Le KY, Dastgheyb S, Ho TV, Otto M. 2014. Molecular determinants of staphylococcal biofilm dispersal and structuring. Frontiers in Cellular and Infection Microbiology 4.

21. Cramton SE, Gerke C, Schnell NF, Nichols WW, Gotz F. 1999. The intercellular adhesion (ica) locus is present in *Staphylococcus aureus* and is required for biofilm formation. Infect Immun 67:5427-33.

22. Pozzi C, Waters EM, Rudkin JK, Schaeffer CR, Lohan AJ, Tong P, Loftus BJ, Pier GB, Fey PD, Massey RC, O'Gara JP. 2012. Methicillin resistance alters the biofilm phenotype and attenuates virulence in *Staphylococcus aureus* device-associated infections. PLoS Pathog 8:e1002626.

23. Boles BR, Horswill AR. 2008. Agr-mediated dispersal of *Staphylococcus aureus* biofilms. PLoS Pathog 4:e1000052.

24. Thomer L, Schneewind O, Missiakas D. 2013. Multiple ligands of von Willebrand factor-binding protein (vWbp) promote *Staphylococcus aureus* clot formation in human plasma. J Biol Chem 288:28283-92.
